# Topologically Associating Domains and Regulatory Landscapes in Development, Evolution and Disease

**DOI:** 10.3389/fcell.2021.702787

**Published:** 2021-07-06

**Authors:** Juan J. Tena, José M. Santos-Pereira

**Affiliations:** Centro Andaluz de Biología del Desarrollo, Consejo Superior de Investigaciones Científicas/Universidad Pablo de Olavide, Seville, Spain

**Keywords:** topologically associating domains, evolution, chromatin structure, genetic diseases, evolutionary novelties, regulatory landscapes

## Abstract

Animal genomes are folded in topologically associating domains (TADs) that have been linked to the regulation of the genes they contain by constraining regulatory interactions between *cis*-regulatory elements and promoters. Therefore, TADs are proposed as structural scaffolds for the establishment of regulatory landscapes (RLs). In this review, we discuss recent advances in the connection between TADs and gene regulation, their relationship with gene RLs and their dynamics during development and differentiation. Moreover, we describe how restructuring TADs may lead to pathological conditions, which explains their high evolutionary conservation, but at the same time it provides a substrate for the emergence of evolutionary innovations that lay at the origin of vertebrates and other phylogenetic clades.

## Introduction

In the last years, the development of chromosome conformation capture (3C) techniques, together with remarkable advances in live cell imaging, have expanded our knowledge about the structural organization of animal genomes (Lieberman-Aiden et al., 2009; McCord et al., 2020; Jerkovic’ and Cavalli, 2021). 3C techniques consist of restriction enzyme digestion of crosslinked chromatin, followed by proximity ligation to generate chimeric molecules that are interpreted as interactions between two genomic regions (McCord et al., 2020). HiC is the genome-wide version of 3C techniques and its increasing resolution has allowed to discern different levels of 3D folding at different scales. At the megabases scale, gene-rich transcriptionally active regions tend to interact among them, while gene-poor heterochromatic regions also interact more frequently, leading to A and B compartments, respectively. At the sub-megabase scale, chromatin domains with high interaction frequency and relatively isolated from neighbor regions form topologically associating domains (TADs). Finally, below the scale of TADs, chromatin loops are formed by strong interactions between specific genomic regions, i.e., CCCTC-binding factor (CTCF) and enhancer-promoter loops (Bonev and Cavalli, 2016; Rada-Iglesias et al., 2018; Rowley and Corces, 2018).

Topologically associating domains are believed to facilitate interactions between *cis*-regulatory elements (CREs) and their target promoters, which otherwise would not occur at enough frequency to ensure a robust target gene expression (Galupa and Heard, 2017; Franke and Gomez-Skarmeta, 2018; Furlong and Levine, 2018). Therefore, TADs have been proposed as structural scaffolds for regulatory landscapes (RLs; Acemel et al., 2017), which are defined as “large genomic regions containing several long-range-acting regulatory sequences that control one or several target genes in a coordinated manner” (Spitz et al., 2003; Bolt and Duboule, 2020). However, whether TADs represent a privileged functional level in the chromosome folding hierarchy has been challenged by the increasing resolution of HiC assays that have uncovered nested structures at the subTAD level with relative insulation among them (Rao et al., 2014; Zhan et al., 2017; Hsieh et al., 2020; Krietenstein et al., 2020). In this review, we discuss the connections of the 3D genome with gene expression, the relationship between TADs and RLs, and their dynamics in the context of development, focusing on disease and evolution.

## Formation of TADs by Loop Extrusion

TAD boundaries are enriched for the binding of CTCF (Phillips and Corces, 2009; Dixon et al., 2012; Merkenschlager and Nora, 2016), an 11-zinc-finger DNA binding protein that was previously known by its role in transcriptional insulation (Filippova et al., 1996; Bell et al., 1999). CTCF is distributed throughout the genome not only at TAD boundaries, but also at many other sites. However, its binding at boundaries has preference for a specific orientation, with its DNA binding motifs often positioned in convergent orientation between the two boundaries that define a TAD (Rao et al., 2014). CTCF co-localizes and determines the presence at TAD boundaries of the cohesin complex, which is also involved in establishing chromosomal interactions (Parelho et al., 2008; Hadjur et al., 2009). These latter observations led to the proposal of the so called “loop extrusion” model for TAD formation involving cohesin and CTCF (de Wit et al., 2015; Sanborn et al., 2015; Fudenberg et al., 2016). According to this model, TADs would arise by chromatin extrusion mediated by cohesin and counteracted by CTCF-mediated insulation, thus explaining both the increased interaction frequency within TADs and their relative insulation from neighbor TADs. Indeed, a recent study has shown that the N-terminal domain of CTCF is essential for blocking cohesin translocation from the interior of TADs, providing a molecular basis for the requirement of a specific binding polarity of CTCF for chromosome folding (Nora et al., 2020). The predictions of this model of TAD formation have been corroborated by the acute depletion of cohesin and CTCF in mammalian cells, which shows loss of chromatin loops and TAD insulation (Nora et al., 2017; Rao et al., 2017), as well as the depletion of factors regulating cohesin loading on chromatin, which leads to differences in the length of loops formed (Busslinger et al., 2017; Haarhuis et al., 2017; Schwarzer et al., 2017; Wutz et al., 2017).

## Regulatory Landscapes and Chromatin Structure

Regulatory landscapes contain CREs that control the expression of their target genes (Bolt and Duboule, 2020). Different mechanisms have been proposed to explain the transcriptional control mediated by CREs: *tracking*, which implies that the RNA polymerase II would bind to enhancers and track along chromatin synthesizing RNA until it reaches the promoter; *linking*, where transcription factors (TFs) would oligomerize from the enhancer to the promoter; or *looping*, in which factors bound to both sides of the loop (enhancer and promoter) would interact with each other (Furlong and Levine, 2018). The latter mechanism is widely accepted and would be favored by CTCF- and cohesin-dependent chromatin folding and promoted by mediator and TFs as Yin Yang 1, a zinc-finger DNA binding protein that form dimers similarly to CTCF and anchors enhancer-promoter interactions (Kagey et al., 2010; Dowen et al., 2014; Weintraub et al., 2017). Direct evidence of the functionality of enhancer-promoter looping comes from studies in the β-globin locus, where forcing these interactions by artificial zinc fingers leads to gene activation (Deng et al., 2014).

An interesting feature of vertebrate RLs is that enhancers are broadly distributed throughout them and not in a gene-centric manner (Symmons et al., 2014). Indeed, the RLs of developmental genes, such as those encoding for lineage-specific TFs or signaling molecules, are characterized by their large sizes and the abundance of enhancers that confer tissue-specific expression to their target genes, thus explaining their common pleiotropy (Bolt and Duboule, 2020). How do enhancers regulate only their target genes? This may be explained in part by the coincidence of RLs coordinates with those of TADs (Symmons et al., 2014). According to this observation, RLs would be confined within TADs and TAD boundaries would correspond to transitions of regulatory domains, therefore ensuring the contact of enhancers with the appropriate target genes and avoiding promiscuous interference with genes located in neighbor domains (see below). However, restriction of RLs within TADs is not enough for gene activation and other mechanisms may influence the outcome of enhancer-promoter contacts (Serebreni and Stark, 2021), including phase separation, which refers to local microenvironments resulting from weak multivalent interactions that concentrate some factors and exclude others (Banani et al., 2017), and enhancer-promoter compatibility (see below).

The RL of the *Shh* gene, encoding an important morphogen involved in the patterning of the developing neural tube and limb buds, is one of the best studied cases in vertebrates. This gene is located within an evolutionary conserved TAD of around 1 Mb in size that contains other non-related genes and multiple tissue-specific enhancers (Lettice et al., 2003; Sagai et al., 2005, 2009; Jeong et al., 2006). In particular, the enhancer known as ZRS [zone of polarizing activity (ZPA) regulatory sequence] is responsible for *Shh* expression in the limb bud and is located around 900-kb away, in the intron 5 of the *Lmbr1* gene (Lettice et al., 2003). Disruption of the ZRS sequence in mice causes loss of limbs, while in humans point mutations cause preaxial polydactyly (Sagai et al., 2005). Other paradigmatic examples of vertebrate RLs are the loci containing the *HoxD* and *HoxA* gene clusters, homeobox genes that are largely responsible for the patterning of several body structures including limbs. These clusters are located between two adjacent TADs that compartmentalize long-range regulatory interactions in two blocks at the spatial and temporal levels (Lonfat and Duboule, 2015). Thus, enhancers in the 3′ TAD preferentially contact “anterior” *Hox* genes, while enhancers in the 5′ TAD mostly interact with “posterior” *Hox* genes (Lonfat et al., 2014). In addition, a switch in the interactions of central genes explains a sequential transition between two regulatory phases (Andrey et al., 2013). The regulatory activity of these enhancers is therefore combined to generate the collinearity of *Hox* genes. Apart from these well-known examples, other cases have been studied and recently reviewed (Bolt and Duboule, 2020).

## The Link Between Chromatin Structure and Function

**FIGURE 1:**
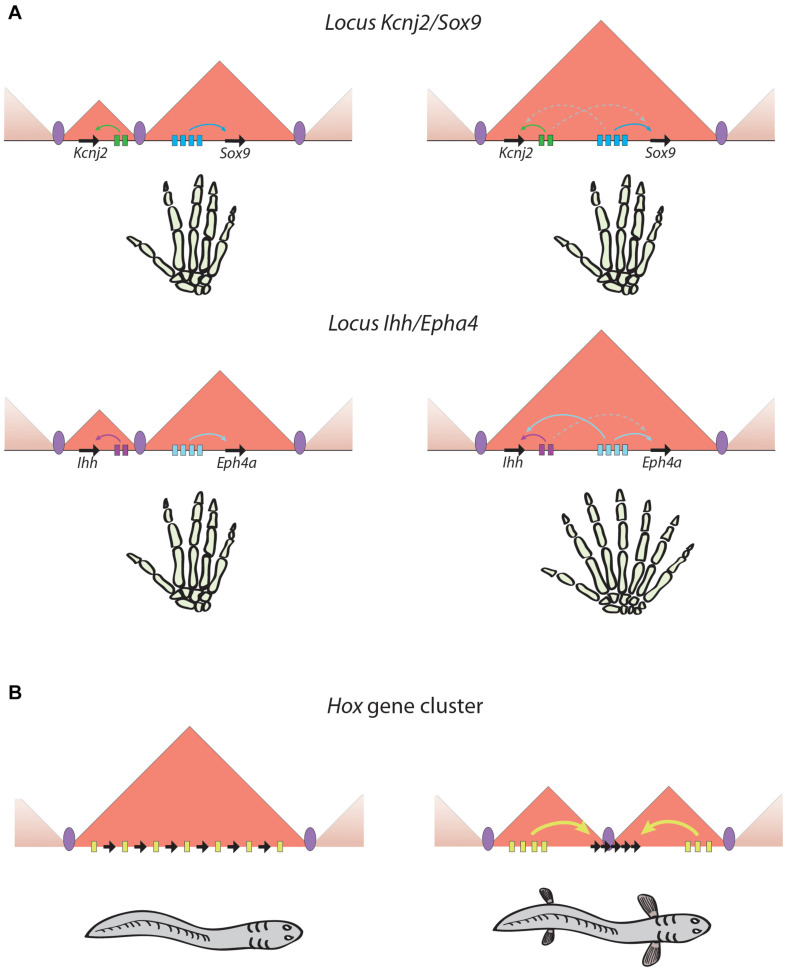
Phenotypical outputs of regulatory landscapes rearrangements. **(A)** Similar rearrangements may lead to very different situations. In the two examples illustrated in this panel, the fusion of two adjacent TADs produces no detectable phenotype in the case of the *Kcnj2/Sox9 locus* (Despang et al., 2019), and a striking polydactylia phenotype in the case of the *Ihh/Eph4a locus* (Lupiáñez et al., 2015). **(B)** TAD restructuring can generate evolutionary innovations by gaining new regulatory inputs or by increasing regulatory complexity. An example is the splitting of the *HoxD* and *HoxA* ancestral TAD found in non-vertebrate chordates in two TADs that are vertebrate-specific and could favor the emergence of vertebrate appendages (Acemel et al., 2016). Black arrows, genes; colored rectangles, enhancers; colored arrows, productive enhancer-promoter interactions; dashed arrows, unproductive enhancer-promoter interactions; purple ellipses, TAD boundaries.

The relationship between TADs and gene expression remains an issue of open debate due to apparent discrepancies between different approaches. Studies analyzing structural variations encompassing TAD boundaries have provided strong links between the chromatin architecture of particular loci and the expression of the nearby developmental genes, which become miss-expressed causing developmental abnormalities (Spielmann et al., 2018; Ibrahim and Mundlos, 2020). Although the adoption of enhancers by genes that were not previously in contact with them explains some of these phenotypes (Lupiáñez et al., 2015; Franke et al., 2016), this is not always the case (Laugsch et al., 2019) and the effects of structural variations on gene expression are context-specific (Figure 1A), which suggests that additional mechanisms may be involved. Indeed, fusion of neighbor TADs by boundary removal at the *Sox9-Kcnj2* locus does not result in major effects on gene expression (Despang et al., 2019). A study in *Drosophila* using highly rearranged balancer chromosomes concluded that TAD rearrangements did not result in altered expression of most genes (Ghavi-Helm et al., 2019). In agreement with this, the works describing alterations in chromatin structure and boundary deletions in the *Shh* locus show minor consequences on *Shh* expression not leading to developmental phenotypes (Paliou et al., 2019; Williamson et al., 2019).

The absence of transcriptional effects observed in some cases of TAD disruption may be explained by the lack of compatibility between enhancers and promoters. In this sense, it has been reported that transcriptional cofactors are able to activate only specific core promoters (Haberle et al., 2019), and that a particular group of highly conserved developmental enhancers, known as poised enhancers, contain CpG islands and are able to activate only developmental promoters also harboring CpG islands (Pachano et al., 2020). Moreover, it has been shown that enhancers within genomic regulatory blocks, which are regions containing a number of highly conserved non-coding elements, only activate particular target genes and not syntenic bystander genes (Akalin et al., 2009). While target genes commonly encode for developmental TFs and have promoters showing long CpG islands and multiple TF motifs, bystander genes often encode for proteins involved in unrelated functions, have different expression patterns and contain promoters with short CpG islands and few TF motifs. These observations indicate that enhancer-promoter compatibility mechanisms may determine the consequences of TAD restructuring at the transcriptional level.

On the other hand, several studies have attempted to remove architectural proteins and assess the effects of their loss genome wide. In this sense, the acute depletion of cohesin or CTCF in mammalian cells shows only a moderate effect in gene expression, affecting from some hundreds to few thousands of genes in different systems (Nora et al., 2017; Rao et al., 2017; Kubo et al., 2021). These studies have been limited *in vivo* due to the essential nature of cohesin and CTCF (Moore et al., 2012; Ju et al., 2013); however, this limitation was recently overcome by generating zebrafish *ctcf* knock-out embryos (Franke et al., 2020). In these embryos, a prolonged maternal contribution allows the survival of the mutant embryos until larval stages, when the absence of CTCF results in the miss-regulation of thousands of genes enriched in developmental functions (Franke et al., 2020). Although part of the effects seen in CTCF depletion or knock-out approaches may be indirect due to CTCF function as a TF, a subset of chromatin interactions involving lineage-specific genes change upon CTCF loss (Kubo et al., 2021). These data suggest that chromatin architecture might not be essential for the expression of most genes, but instead provide robustness to the expression of developmental genes that are frequently regulated by many CREs within complex RLs.

## Chromatin Structure Dynamics During Development and Differentiation

An important question in the field of chromatin structure is how variable are TADs during dynamic processes, including embryonic development or cell differentiation. Studies of chromatin structure during animal early development have revealed a phase in which TADs become undetectable followed by a progressive reestablishment of chromatin folding (van der Weide and de Wit, 2019). However, there are some differences between species regarding when this phase happens in relation to the zygotic genome activation (ZGA). While in mammals the period without detectable chromatin structure takes place before the ZGA, with structure being progressively reestablished from there (Du et al., 2017; Gassler et al., 2017; Chen et al., 2019), similar to *Drosophila* (Hug et al., 2017), in zebrafish and medaka embryos it occurs during the ZGA and persist after it, being reestablished mostly during gastrulation (Kaaij et al., 2018; Nakamura et al., 2018), but there are observed discrepancies regarding the existence of chromatin structure before the ZGA in zebrafish (Kaaij et al., 2018; Wike et al., 2021). Although once formed, TADs seem to be very stable at later developmental stages, little is known about the correlation of these structures with progressive changes in gene expression.

Several studies have reported that TADs are largely stable when compared between embryonic stem cells and differentiated cells (Dixon et al., 2012, 2015; Nora et al., 2012). Consistent with this observation, a recent study has shown that chromatin structure in *Drosophila* embryos is preserved in different embryonic tissues despite lineage-specific gene expression (Ing-Simmons et al., 2021). However, another report showed a high variability of TAD boundaries across 37 human cell types (McArthur and Capra, 2021). It is worth noting that these apparent discrepancies might be explained either by the different biological systems used or by different resolutions in HiC experiments, as well as different computational methods to call TADs or TAD boundaries. At lower resolutions than TADs, it was shown that variations in intra-TAD contacts during mammalian differentiation corresponded with switches between active and inactive chromatin modifications and gene expression (Dixon et al., 2015). Indeed, enhancer-promoter interactions are highly dynamic and cell-type-specific during neural and erythroid differentiation, accompanying the activation of lineage-specific genes (Bonev et al., 2017; Oudelaar et al., 2020; Kubo et al., 2021). However, enhancer-promoter interactions are stably formed before gene activation in other contexts. Indeed, poised enhancers required for the activation of anterior neural genes are already engaged in contacts with their target genes in mESCs in a polycomb repressive complex 2-dependent manner (Cruz-Molina et al., 2017). However, enhancer-promoter loops in *Drosophila* precede target gene transcription and even TAD formation, being associated with paused RNA polymerase (Ghavi-Helm et al., 2014; Espinola et al., 2021). These observations suggest that the dynamics or stability of TADs and enhancer-promoter loops are highly context-specific, some of them being stable and others being dynamically regulated during development and differentiation.

## Alterations of the 3D Genome Associated With Disease

Strong evidence for the importance of genome architecture for correct gene expression comes from studies showing that the disruption of 3D structure in particular loci, either by genomic rearrangements or alterations in TAD boundaries, lead to pathological situations, including developmental disorders or cancer (Ibrahim and Mundlos, 2020). A number of cases reported in the last years have provided a link between structural variations and disease. At the *Epha4* locus, deletions, duplications and inversions disrupting TAD structure cause several limb malformations, including brachydactyly, polydactyly and F-syndrome, due to *de novo* enhancer-promoter interactions that lead to gene miss-expression of the surrounding genes *Wnt6*, *Ihh* and *Pax3* (Figure 1A; Lupiáñez et al., 2015). At the *Sox9* locus, duplications encompassing the neighbor *Kcnj2* gene lead to the formation of a “neo-TAD” in which *Kcnj2* is miss-regulated by interactions with new enhancers, leading to a limb malformation phenotype known as Cooks syndrome (Franke et al., 2016). A similar situation has been reported in the locus of *GDPD1* gene in autosomal-dominant retinitis pigmentosa, caused by a rearrangement that place a TAD border and several retinal enhancers within this locus. This generates a “neo-TAD” and new contacts between the retinal enhancers and *GDPD1* gene, which is overexpressed and likely contributes to the disease (de Bruijn et al., 2020). Finally, inversions at the *TFAP2A* locus in branchiooculofacial syndrome patients have been shown to disconnect this gene from its neural crest-specific enhancers, leading to its reduced expression and explaining the patient’s phenotype (Laugsch et al., 2019).

Genomic rearrangements can also lead to the fusion of genes that become overexpressed and function as oncogenes. This is the case of chromosomal translocations fusing the genes *PAX3* and *FOXO1* in alveolar rhabdomyosarcoma, which result in the fusion of both RLs and the activation of transcription from *PAX3* promoter by enhancers from the *FOXO1* RL (Vicente-Garcia et al., 2017). At larger scales, it has been found that higher order chromatin-folding structures can modulate interactions between different loci spanning several megabases in a highly aggressive type of squamous cell carcinoma (Alekseyenko et al., 2015; Rosencrance et al., 2020). The appearance of these interactions, called “megadomains,” responds to the formation of large regions of hyperacetylated chromatin due to the BRD4-NUT fusion oncoprotein. These fusions are generated by genomic translocations, usually between genes like *BRD4* or *BRD3* and *NUT*, which recruits the histone acetyltransferase p300 leading to hyperacetylated regions of up to 2 Mb. Both intra- and interdomain interactions are up-regulated, as well as the expression of the oncogenes *SOX2*, *TP63* and *MYC*, which eventually contribute to tumorigenesis (Alekseyenko et al., 2015; Rosencrance et al., 2020; Eagen and French, 2021).

Apart from genomic rearrangements, alterations of TAD boundaries have also been linked to disease. The analyses of copy-number variants involving TAD boundary deletions revealed that a substantial proportion of cases could be explained by enhancer adoption (Ibn-Salem et al., 2014). In a recent work, CTCF binding sites surrounding the well-known *ZRS* enhancer of the *Shh* gene (see above) were removed by CRISPR-mediated genome editing in mice. Strikingly, the lack of these CTCF binding sites reduced the interaction between *ZRS* and *Shh* promoter, as well as the expression of the latter (Paliou et al., 2019). Nevertheless, this does not lead to a clear phenotype in these mice unless a hypomorphic allele of the ZRS is used, suggesting that chromatin structure provides robustness but does not determine enhancer-promoter communication. Indeed, a recent study from The Cancer Genome Atlas showed that only 14% of cancer-associated TAD boundary deletions resulted in significant changes in expression of the nearby genes (Akdemir et al., 2020). Moreover, mutations in CTCF binding sites have been frequently found in cancer (Katainen et al., 2015), which might lead to miss-expression of nearby genes causing tumorigenesis. Indeed, the disruption of boundaries demarcating insulated neighborhoods, which are chromatin domains smaller in size than TADs, leads to aberrant activation of proto-oncogenes, such as *TAL1* and *LMO2* associated with T-cell acute lymphoblastic leukemia (Hnisz et al., 2016). In the opposite situation, the overexpression of the Neurotensin gene *NTS*, a central nervous system neurotransmitter, has been recently related with melanomas due to a gained CTCF-mediated chromatin loop that establish contacts between the *NTS* promoter and a CRE in the intron of the *LRRIQ1* gene located 800 Kb away (Chai et al., 2021).

It is worth to remark that mutations in the coding sequence or miss-expression of architectural proteins, i.e., cohesin and CTCF, can lead to a wide set of human pathological phenotypes (Singh and Gerton, 2015). For example, somatic mutations in cohesin have been associated with different forms of cancer (Mullenders et al., 2015; Fisher et al., 2017; Carico et al., 2021), which is also related to the important role of cohesin in the separation of sister chromatids during cell division (Nasmyth and Haering, 2009). In addition, CTCF has been proposed as a tumor suppressor gene, since mutations in its coding sequence have also been detected in different types of cancer (Filippova et al., 2002). Interestingly, different missense mutations affected specific zinc-finger domains, leading to defects in the binding to the promoters of a subset of genes involved in the regulation of proliferation, but not to others. Therefore, both cohesin and CTCF play an essential role in gene regulation that prevents pathological situations.

## Conservation of TADs Across Genome Evolution

Several studies comparing chromatin structure in diverse species have reached the conclusion that TADs and their boundaries are largely conserved in animal genomes. The first HiC data comparing TADs in human and mouse cells found a general conservation of their boundaries (Dixon et al., 2012) and an in-depth analysis of conservation in mammals revealed that conserved TAD boundaries were associated with conserved CTCF sites, while divergent CTCF sites correlated with divergence of chromatin structure (Vietri Rudan et al., 2015). Indeed, CTCF binding at TAD boundaries is highly clustered and these sites are subjected to stronger selective constrains than other CTCF sites among closely related species (Kentepozidou et al., 2020; McArthur and Capra, 2021). Therefore, the strong selection against disruption of TAD boundaries in evolution is likely responsible for their enrichment in rearrangement breakpoints in vertebrates, being reshuffled as whole blocks during evolution (Krefting et al., 2018). A clear case of this conservation is the boundary splitting the RLs of the *Six* genes, which is conserved not only in vertebrates but also in echinoderms, illustrating the deep conservation of TADs involving important developmental genes (Gomez-Marin et al., 2015). Finally, studies analyzing highly conserved gene regulatory blocks, which are clusters of conserved non-coding elements around important developmental regulators, have revealed that a subset of TADs exhibit extreme non-coding conservation across metazoans (Harmston et al., 2017). Therefore, TADs involving developmental genes represent evolutionary conserved chromatin domains likely because they provide a scaffold for developmental RLs.

## TAD Reshuffling Underlies the Emergence of Evolutionary Novelties

As commented above, genomic rearrangements involving TAD restructuring and the associated alterations in gene expression usually entail deleterious effects. Nevertheless, they also provide a substrate for evolution and changes in genome structure may result in the gain of new functions that underlie the appearance of evolutionary novelties during species evolution (Maeso et al., 2017; Figure 1B). This has probably been the case with the emergence of limbs in vertebrates, for which the restructuring of TADs at the *HoxA* and *HoxD* loci has been essential. As can be inferred from slow evolving, non-vertebrate animals like amphioxus, the ancestral *Hox* locus was organized in a single TAD encompassing all *Hox* genes and the enhancers regulating their expression. However, at the origin of vertebrates this TAD was split in two located at either sides of the cluster and separating some genes from the rest, while leaving some others in the hinge between both TADs (Figure 1B; Acemel et al., 2016). This organization allowed the spatial and temporal segregation of regulatory inputs that explains the collinearity of the *HOX* genes (see above; Andrey et al., 2013; Lonfat et al., 2014) and likely enabled the plasticity in the usage of the Hox patterning system that was essential for vertebrate evolutionary novelties such as the development of paired appendages. On the other hand, the gnathostome-specific expression of *Shh* in the limbs was shown to be originated between the two whole-genome duplications by a translocation that linked the RL of that gene with *Lmbr1* (Irimia et al., 2012). Then, the ZRS enhancer could emerge in the intron 5 of *Lmbr1*, being critical for the emergence of paired and unpaired appendages (Letelier et al., 2018a).

Apart from TAD restructuring early in the evolution of vertebrates, other cases have been documented when comparing relatively close species. Thus, the regulatory cluster formed by *rac3b*, *rfng* and *sgca* genes emerged at the origin of the Ostariophysi fish superorder by a genomic rearrangement that brought in contact the RLs of *rac3b/rfng* and *sgca*, which are separated in Actinopterygii and tetrapods (Letelier et al., 2018b). Such rearrangement led to the formation of new regulatory contacts between *rac3b* and *rfng* promoters and the ancestral *sgca* RL, which was responsible to direct its expression to the hindbrain boundaries. These new regulatory interactions resulted in the co-option (which refers to the redeployment of pre-existing genetic or regulatory mechanisms for the acquisition of new functions or expression domains) of *rac3b* and *rfng* in the rhombomeres margins, thus promoting the formation of actomyosin cables characteristic of these structures (Letelier et al., 2018b). Moreover, it has been found that human brain tissue shows a subset of species-specific TADs compared with macaques that are associated with human-specific expression changes that are likely responsible of the higher complexity of the human brain (Luo et al., 2021). Similarly, interspecies differences in chromatin structure between human and chimpanzees are commonly associated with differences in gene expression (Eres et al., 2019). These differences between phylogenetically related species have also been discovered in two different species of *Drosophila* genus, *D. melanogaster* and *D. triauraria*. Only 25% of the TADs are orthologous between both species, and importantly these genomic rearrangements could be related with changes in gene expression (Torosin et al., 2020). These findings support the hypothesis that reorganization of genomic 3D structure may act as an important force in the rise of evolutionary novelties.

## Concluding Remarks

Although the recent technological advances have allowed an increasingly detailed understanding of the 3D organization of the genome, many questions remain unanswered. How CREs operate within the context of TADs over their target genes in a mechanistic level remains incompletely understood, and to what extent the alterations of TADs lead to gene miss-expression is still an open debate. Moreover, our knowledge of how the restructuring of TADs leads to evolutionary innovations is limited to a few reported examples and the scarce availability of genome-wide chromosome conformation experiments in different species limits the comparative analyses of the 3D genome from an evolutionary perspective. Finally, the increasing applicability of the single cell technologies to chromosome conformation experiments will hopefully make possible to discern between chromatin structure in particular cell types and its association with cell type-specific gene expression during development and differentiation.

## Author Contributions

JS-P and JT reviewed the articles and wrote the manuscript. Both authors contributed to the article and approved the submitted version.

## Conflict of Interest

The authors declare that the research was conducted in the absence of any commercial or financial relationships that could be construed as a potential conflict of interest.
